# Relationship between Influenza Vaccination Coverage Rate and COVID-19 Outbreak: An Italian Ecological Study

**DOI:** 10.3390/vaccines8030535

**Published:** 2020-09-16

**Authors:** Mauro Amato, José Pablo Werba, Beatrice Frigerio, Daniela Coggi, Daniela Sansaro, Alessio Ravani, Palma Ferrante, Fabrizio Veglia, Elena Tremoli, Damiano Baldassarre

**Affiliations:** 1 Centro Cardiologico Monzino, IRCCS, 20138 Milan, Italy; mauro.amato@ccfm.it (M.A.); pablo.werba@ccfm.it (J.P.W.); beatrice.frigerio@ccfm.it (B.F.); daniela.coggi@ccfm.it (D.C.); daniela.sansaro@ccfm.it (D.S.); alessio.ravani@ccfm.it (A.R.); ferrante.palma@cardiologicomonzino.it (P.F.); fabrizio.veglia@ccfm.it (F.V.); elena.tremoli@ccfm.it (E.T.); 2 Dipartimento di Scienze Farmacologiche e Biomolecolari, Università degli Studi di Milano, 20133 Milan, Italy; 3 Department of Medical Biotechnology and Translational Medicine, Università degli Studi di Milano, 20129 Milan, Italy

**Keywords:** influenza vaccination coverage rate, COVID-19, coronavirus spread

## Abstract

*Background:* The lack of specific vaccines or drugs against coronavirus disease 2019 (COVID-19) warrants studies focusing on alternative clinical approaches to reduce the spread of this pandemic disease. In this study, we investigated whether anti-influenza vaccination plays a role in minimizing the diffusion of COVID-19 in the Italian population aged 65 and over. *Methods:* Four COVID-19 outcomes were used: severe acute respiratory syndrome coronavirus 2 (SARS-CoV-2) seroprevalence, hospitalizations for COVID-19 symptoms, admissions to intensive care units for reasons related to SARS-CoV-2, and deaths attributable to COVID-19. *Results:* At univariate analyses, the influenza vaccination coverage rates correlated negatively with all COVID-19 outcomes (Beta ranging from −134 to −0.61; all *p* < 0.01). At multivariable analyses, influenza vaccination coverage rates correlated independently with SARS-CoV-2 seroprevalence (Beta (95% C.I.): −130 (−198, −62); *p* = 0.001), hospitalizations for COVID-19 symptoms (Beta (95% C.I.): −4.16 (−6.27, −2.05); *p* = 0.001), admission to intensive care units for reasons related to SARS-CoV-2 (Beta (95% C.I.): −0.58 (−1.05, −0.12); *p* = 0.017), and number of deaths attributable to COVID-19 (Beta (95% C.I.): −3.29 (−5.66, −0.93); *p* = 0.010). The R^2^ observed in the unadjusted analysis increased from 82% to 159% for all the considered outcomes after multivariable analyses. *Conclusions:* In the Italian population, the coverage rate of the influenza vaccination in people aged 65 and over is associated with a reduced spread and a less severe clinical expression of COVID-19. This finding warrants ad hoc studies to investigate the role of influenza vaccination in preventing the spread of COVID-19.

## 1. Introduction

In December 2019, an outbreak of COVID-19, an atypical pneumonia associated to a novel coronavirus (severe acute respiratory syndrome coronavirus 2; SARS-CoV-2) was detected in Wuhan, Hubei province, China.

Epidemiological data showed that SARS-CoV-2 mainly infects and threatens the health of the elderly and subjects with comorbidities, such as diabetes, obesity, cardiovascular, respiratory, renal, and lung diseases [1]. Due to its high infectiousness, SARS-CoV-2 has spread rapidly throughout the globe, and on 11 March, 2020, the World Health Organization (WHO) officially recognized COVID-19 as a pandemic [2].

Italy has been strongly affected by COVID-19 [3]. The first Italian case was identified on February 21, 2020, at about the same time that the seasonal flu, caused by the influenza virus, reached its annual peak of diffusion [4]. The diseases caused by influenza virus and SARS-CoV-2 both share a similar route of transmission (i.e., through aerosolized or respiratory droplets) and some respiratory and systemic symptoms, but they strongly differ in terms of rates of severe and fatal cases [5], and peculiarly, of the age groups predominantly affected. Indeed, influenza preferentially affects children and young adults [6], whereas the symptomatic SARS-CoV-2 infection rate increases with age, from about 50–100 cases/100,000 in children and subjects younger than 20 to about 900 cases/100,000 in subjects over 80 years old [7,8]. One possible explanation for the low susceptibility in children and young people to the SARS-CoV-2 infection is a more effective and reactive immune system, boosted by the exposure to common pediatric viral agents or to viral antigens contained in the numerous anti-viral vaccines (measles, mumps, rubella, varicella, hepatitis B, hepatitis A, rotavirus, papilloma virus) administered early in life. Indeed, vaccines may induce positive “non-specific” immunotherapeutic mechanisms improving the host’s response to other pathogens, through a process named “trained immunity” [9].

Annual vaccination, a major component of public health programs, is the most efficacious and cost-effective intervention to prevent seasonal influenza [10]. In addition, during the coronavirus SARS-CoV-1 outbreak which occurred in China at the end of 2002, the WHO recommended increasing the coverage rate of influenza vaccination, particularly in frail groups (e.g., elderly and disabled people) [11].

As neither a specific vaccine against SARS-CoV-2 nor anti-COVID-19 drugs are available yet, and since several pieces of evidence suggest a real risk of a second wave of outbreaks [12,13], it may be relevant to assess whether influenza vaccination could mitigate a new COVID-19 outbreak. As a first investigational approach, in the present study we evaluated whether the different influenza vaccination coverage rates reached in people aged 65 and over in each Italian region are associated with a different spread of the COVID-19 outbreak.

## 2. Materials and Methods

### 2.1. Study Design

These ecological study analyses aggregate data of the 19 regions and of the two Autonomous Provinces (AP); i.e., Bozen and Trento from the Trentino Alto Adige region, for a total number of 21 regions and AP considered.

### 2.2. Definition of Outcomes and Predictors

Data of each region and AP were obtained from the Italian Institute of Statistic (ISTAT) [14] and from the Italian Civil Protection Department [15]. From these free data sources, it was possible to collect data on four types of outcomes related to confirmed COVID-19 cases: (1) the SARS-CoV-2 seroprevalence, (2) the number of patients hospitalized, (3) the number of patients admitted to intensive care units, and (4) the number of deaths attributable to COVID-19. The SARS-CoV-2 seroprevalence of each region and AP was obtained from the ISTAT report [14]. Concerning the other COVID-19 outcomes, data were collected between 10 March 2020 and 2 June 2020; i.e., the period corresponding to the national lockdown imposed by the Italian government to limit the outbreak. As the absolute value of these variables depends on the total number of subjects living in the region, data are expressed as number of cases per 100,000 inhabitants in each region and AP. To obtain these figures, the number of cases was divided by the total number of subjects living in the region and AP on 1 January 2019 [16], and then multiplied by 100,000. All outcomes refer to the entire population of Italy.

### 2.3. Influenza Vaccination Coverage

Official data on the prevalence of the influenza vaccination coverage rate among subjects aged 65 and over were obtained from the website of the Italian Ministry of Health [17]. Since the data on regional influenza vaccination coverage rates for the 2019–2020 season were not available yet, for the purpose of this ecological analysis, these data were extrapolated by a linear regression equation considering the regional trend of the influenza vaccination coverage rate over the last five years (seasons 2014–2015 to 2018–2019) (Figure S1).

### 2.4. Confounders

As possible confounders of the relationships between the regional influenza vaccination coverage rates and the aforementioned outcomes, we considered seven variables suggested as predictors of COVID-19 spread: (1) the percentage of health expenditures with respect to the regional Gross Domestic Product (GDP) [18], (2) the mean seasonal temperature of each specific region and AP [19,20,21], (3) the delay in applying the lockdown [22], (4) the import–export commerce between Italy and China [23], (5) the international air traffic [24], (6) the mortality from cardiovascular diseases [25,26], and (7) the mortality from respiratory diseases [27]. The percentages of health expenditures with respect to GDP were obtained from the 2019 Italian report from the Observatory on Healthcare Organizations and Policies in Italy (OASI) [28]. The mean seasonal temperature of each region was estimated considering the coldest season (i.e., from 1 November 2019 to 31 March 2020) and computed as the average of the mean monthly temperature of the monitored cities [29] of each region. The delay in each region and AP in implementing the lockdown was indexed in terms of the number of days between the first date with a net reproduction number “Rt” > 1 (i.e., the day when the mean number of new secondary infections caused by a primary infected patient increased above the epidemic threshold of 1, thus indicating the beginning of the infection spread) and the date when the human mobility flows decreased by at least 20%, as compared to the mobility flows detected in the pre-epidemic period (i.e., from 1 January 2020 to 16 February 2020) [22]. The financial relationship between each Italian region and AP and China was estimated by using data on import and export commerce (in millions of Euros) between each Italian region and China obtained by the ISTAT [30]. The air traffic volume was quantified by considering the total number of passengers on international flights that passed through all the airports of each Italian region in the last months before the flight blockade (i.e., from October 2019 to March 2020) [31]. The regional mortality rates from cardiovascular diseases and from respiratory diseases were obtained by ISTAT (last update: 2017) and expressed in terms of number of cases per 100,000 inhabitants [30].

### 2.5. Ethics Committee Approval

Given the ecological nature of the study and the use of publicly available aggregated data, no ethical approval was needed.

### 2.6. Statistical Analysis

Crude and adjusted regional predictors of the considered outcomes were assessed by Pearson’s regression and by multiple linear regression analyses. In the multiple linear regression analysis performed to identify COVID-19 independent predictors, each COVID-19 outcome was set a priori as the dependent variable, whereas the regional vaccination coverage rate and the aforementioned confounders that were significant at univariate analysis were set as independent variables. R^2^ value was used to determine the goodness of fit of the linear models in the prediction of COVID-19 outcomes. All analyses were performed with SPSS 25.0 for Windows (SPSS, Inc., Chicago, IL, USA). *p* values < 0.05 were considered statistically significant.

## 3. Results

### 3.1. Influenza Vaccination Coverage Rate and COVID-19 Outcomes

**Figure 1:**
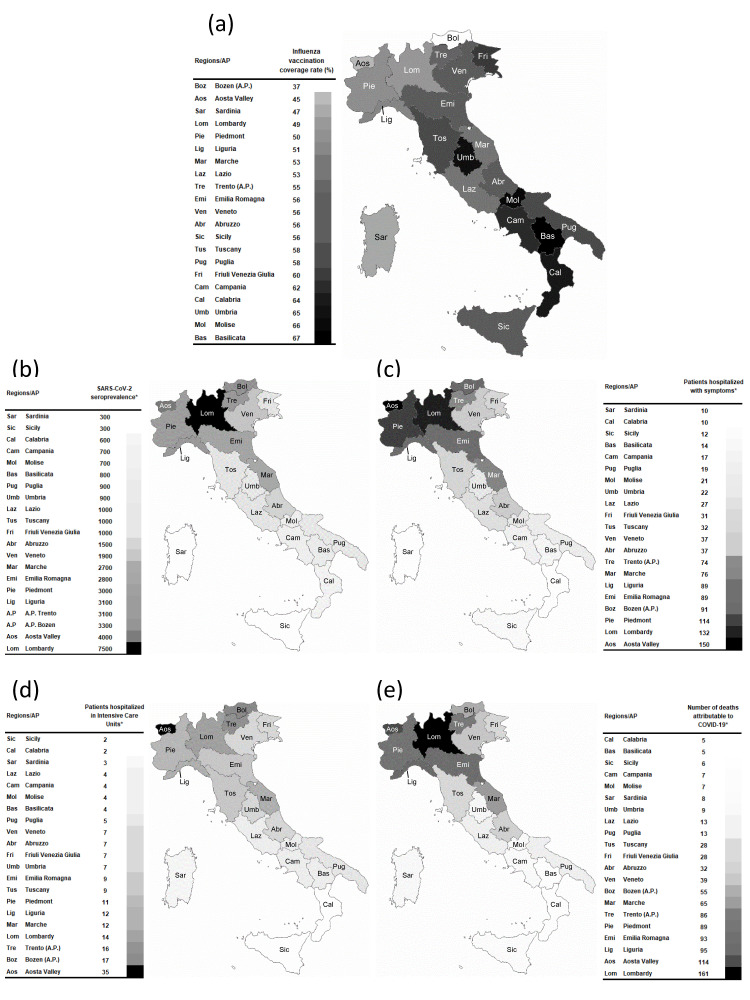
(**a**) Influenza vaccination coverage rate, (**b**) seroprevalence for COVID-19, (**c**) patients hospitalized with symptoms, (**d**) patients hospitalized in intensive care units, and (**e**) number of deaths attributable to COVID-19 in the regions/AP. * cases/100,000 inhabitants.

Data on influenza vaccination coverage rate (panel a), SARS-CoV-2 seroprevalence (panel b), and occurrence of COVID-19 clinical outcomes (per 100,000 inhabitants) in each region and AP (panels c, d, and e) are shown in Figure 1 and Table S1. Influenza vaccination coverage rates were highly variable among the regions and AP, ranging from 37% in the autonomous province of Bozen to 67% in Basilicata.

**Figure 2:**
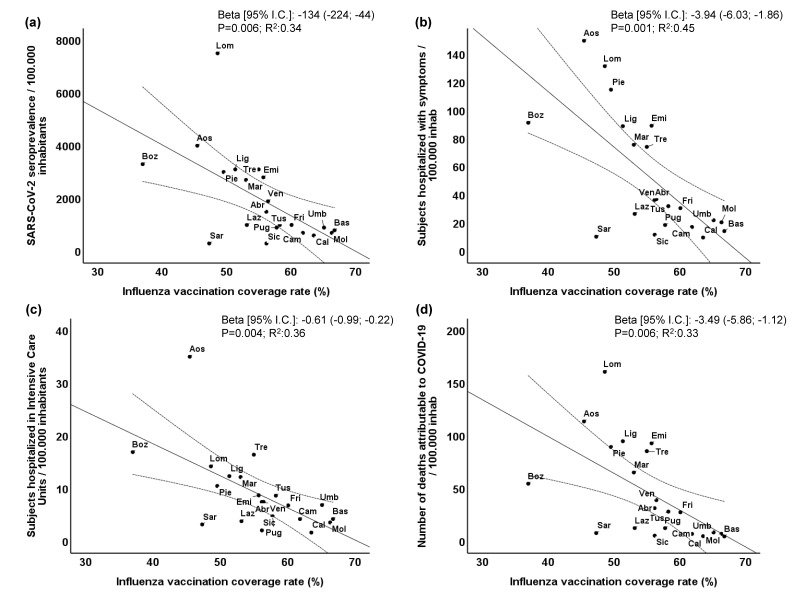
Univariate correlations between the influenza vaccination coverage rate and (**a**) seroprevalence for COVID-19, (**b**) patients hospitalized with symptoms, (**c**) patients hospitalized in intensive care units or (**d**) number of deaths attributable to COVID-19. Abr = Abruzzo; Aos = Aosta Valley; Bas = Basilicata; Boz = Bozen A.P.; Cal = Calabria; Cam = Campania; Emi = Emilia Romagna; Fri = Friuli Venezia Giuli; Laz = Lazio; Lig = Liguria; Lom = Lombardy; Mar = Marche; Mol = Molise; Pie = Piedmont; Pug = Puglia; Sar = Sardinia; Sic = Sicily; Tre = Trento A.P.; Tus = Tuscany; Umb = Umbria; Ven = Veneto.

Inverse and significant univariate correlations along the different regions and AP between the influenza vaccination coverage rate and the SARS-CoV-2 seroprevalence (panel a), patients hospitalized with symptoms (panel b), patients hospitalized in intensive care units (panel c), and number of deaths attributable to COVID-19 (panel d) were found (Figure 2). All the dependent variables were normalized per 100,000 inhabitants. The Beta correlation coefficient ranged from −134 to −0.61 (all *p* ≤ 0.01). The R^2^ values ranged from 0.33 for number of deaths attributable to COVID-19 to 0.45 for patients hospitalized with symptoms.

### 3.2. Role of Potential Confounders

The values of the potential confounders used in the multiple linear regression analyses are indicated in Table S1. Among the seven potential confounders considered (see Materials and Methods), the public health expenditure associated positively with influenza vaccination coverage rate (*r* = 0.54; *p* < 0.05) and negatively with all COVID-19 outcomes (r values ranging from −0.72 to −0.60; all *p* < 0.01) (Table 1). Mean seasonal temperature associated negatively with all COVID-19 outcomes (all *p* < 0.01). The delay in applying the lockdown and the import–export exchanges with China correlated positively with the SARS-CoV-2 seroprevalence (both *p* < 0.05), with the number of patients hospitalized with symptoms (both *p* < 0.05), and with the number of deaths attributable to COVID-19 (*p* < 0.05 and *p* < 0.01, respectively). The mortality rate for cardiovascular diseases correlated positively with the influenza vaccination coverage rate and negatively with SARS-CoV-2 seroprevalence (*r* = 0.61 and *r* = −0.53, respectively; both *p* < 0.05). The international air traffic and the mortality from respiratory diseases did not correlate significantly with any COVID-19 outcome, and therefore they were not included among the possible confounders in the multivariate analyses.

Table 2 shows Beta and 95% C.I. of the relationships between the influenza vaccination coverage rate and COVID-19 outcomes, after adjustment for the five covariates and potential confounders significantly associated with at least one COVID-19 outcome at univariate analysis. The influenza vaccination coverage rate was an independent predictor of SARS-CoV-2 seroprevalence (*p* = 0.001), COVID-19-related hospitalization (*p* = 0.001), hospitalization in intensive care units (*p* = 0.017), and death attributable to COVID-19 (*p* = 0.010). Concerning the role of potential confounders themselves, we found that the import–export with China was positively associated with the SARS-CoV-2 seroprevalence (*p* < 0.0001), with COVID-19 related hospitalization (*p* = 0.032), and with COVID-19-related deaths (*p* = 0.004), whereas the mortality from cardiovascular diseases was positively associated with COVID-19 related hospitalizations (*p* = 0.029).

## 4. Discussion

Using aggregated data of Italian regions and AP obtained from official national websites, in this ecological study, we found an inverse association between the extent of influenza vaccination coverage rate and the seroprevalence of SARS-CoV-2, the prevalence of patients hospitalized, admitted to intensive care units, or the number of deaths attributable to COVID-19. The strength of these associations, observed at univariate analyses, markedly increased after adjustment for potential confounders, such as the percentage of health expenditures with respect to the regional Gross Domestic Product, the mean seasonal temperature, the delay in applying the lockdown, the Italy–China import–export commerce, and the cardiovascular mortality rate. Indeed, compared with univariate analyses, the adjustment for confounding factors increased the model’s R^2^ by about 159% considering SARS-CoV-2 seroprevalence, ~82% considering patients hospitalized with symptoms, ~94% considering patients hospitalized in intensive care units, and ~136% considering the number of deaths attributable to COVID-19 (Table S2). Most importantly, based on adjusted regression coefficients (Table 2), it can be estimated that a 1% increase in the vaccination coverage rate among subjects aged 65 and over (i.e., about 140,000 doses of vaccine throughout Italy) would have resulted, in the entire Italian population (60.36 million inhabitants), in a reduction of 78,560 seropositive subjects, 2512 hospitalized patients with symptoms, 353 patients hospitalized in intensive care, and 1989 deaths.

To the best of our knowledge, only a few studies have examined the relationship between influenza vaccination and COVID-19 outcomes [32,33,34]. In an ecological study carried out at a county-level in the American elderly population, an inverse association between influenza vaccination coverage rate and deaths attributable to COVID-19 was reported [33]. A similar association was found in an Italian ecological study, although through performing an unadjusted analysis [34]. In our study, we corroborate and extend these observations by showing, in the Italian population, a potential protective role of influenza vaccination on COVID-19 mortality also after adjusting the data for a variety of potential confounders. In addition, we found that influenza vaccination coverage rate is also independently associated with the SARS-CoV-2 seroprevalence and the occurrence of non-fatal clinical expressions of COVID-19, indexed by the rates of hospital admissions and intensive care unit admissions. Our data are in line with a recent Brazilian study [32] reporting a significantly lower risk of hospitalizations in intensive care treatment, of invasive respiratory support, and of death in COVID-19 patients who had recently received influenza vaccination than in those who had not.

Although our findings do not allow for assumptions to be made about the mechanisms underlying the putative protective role of influenza vaccination on COVID-19 outcomes, previous immunological and epidemiological studies [35,36,37,38,39], as well as mathematical models [40,41,42], support the concept that vaccination against one microorganism may affect the host’s response to other infectious agents. For example, vaccination against mycobacterium tuberculosis with the bacillus Calmette–Guérin vaccine significantly increases the secretion of IL-1B (a pro-inflammatory cytokine), which plays a recognized role in antiviral immunity [43].

Alternatively, preventing influenza through vaccination might reduce the risk of respiratory superinfection with SARS-CoV-2. However, a recent report about the rates of co-infection between SARS-CoV-2 and other respiratory pathogens suggests that influenza virus and SARS-CoV-2 co-infection is extremely rare (0.9%) [44]. Yet, this coinfection might determine an enhanced COVID-19 severity, as was found in more than 20% of patients dead from SARS-COV-2 in Northeastern Iran [45]. Finally, people who agree to undertake influenza vaccination may merely have a more proactive attitude towards prevention, including a respect of social distancing, proper use of face masks, and the use of other personal protection equipment (PPE) during the pandemic compared to non-vaccinated people, which could have determined less COVID-19 outcomes in groups with a high rate of influenza vaccination coverage.

Our incidental observation at univariate analyses of a direct correlation between regional influenza vaccination coverage rate and mortality from cardiovascular diseases is counterintuitive and should be interpreted with caution. Indeed, a variety of studies have indicated that influenza vaccination is an effective intervention to reduce cardiovascular risk [46,47,48], and guidelines endorse its administration, mainly in secondary cardiovascular prevention or other conditions of frailty [49]. Therefore, we believe that one possible explanation for the observed association may be “reverse causality” (i.e., more people are vaccinated against seasonal influenza in regions with a higher prevalence of frail individuals, as revealed by a higher cardiovascular mortality rate).

Not surprisingly, in this study, the regional mortality from cardiovascular diseases was positively and independently associated with the regional rate of hospitalizations for COVID-19, a finding in line with numerous reports showing cardiovascular disease as a predisposing factor to symptomatic and more severe clinical expressions of COVID-19 [25,26].

This study has several strengths. First, we used aggregated data from official national web sites, which allows for generalizing our findings to the whole Italian population. Second, having used national level data, our analyses have eluded the effect of potential confounding transnational differences (e.g., cultural norms, medical care standards, health system policy, government response in duration of quarantine or timeliness of the start of the blockade, and homogeneity of data collected), which, in absence of a statistical adjustment [50], might provide spurious associations.

This study also has some limitations. First, as the 2019–2020 regional influenza vaccination coverage rate was not available at the time of our study, our analyses are based on an extrapolation of data of the last five years (see Figure S1). Second, despite our attempt to control for the effects of some of the most important confounders identified in the literature, we cannot exclude an influence on the results of other confounders that have not been identified yet. Third, regional rather than individual data have been used. Therefore, we cannot exclude the ecological fallacy (i.e., the fact that the observed associations might not persist at an individual level) [51]. Fourth, Italian data cannot be automatically extrapolated to other countries.

Pending corroboration through experimental clinical studies with proper design, the results of our ecological analyses might have major public health implications. Indeed, they suggest that the influenza vaccination coverage rate of the population, on top of the currently recommended measures to limit contagion (social distancing, confinement, use of PPE), might contribute to attenuate the SARS-CoV-2 pandemic.

## 5. Conclusions

In conclusion, in the Italian population aged 65 and over, the regional influenza vaccination coverage rate is inversely associated with indices of the SARS-CoV-2 spread and clinical consequences.

Given that influenza vaccination is a safe intervention already recommended by the Italian Health Service for people aged 65 and over, our data are in favor of enhancing influenza vaccination coverage (at least in this segment of the population, which is currently 37 to 67%) to achieve the recommended influenza vaccination coverage rate [11], and warrant further investigations to assess its efficacy as an adjuvant intervention in the fight against the COVID-19 pandemic.

## Figures and Tables

**Table 1:** Pearson’s correlation analysis between possible confounders and influenza vaccination coverage rate and COVID-19 outcomes.

Regional Features/Variables	Influenza Vaccination Coverage Rate	SARS-CoV-2 Seroprevalence	Patients Hospitalized with Symptoms	Patients Hospitalized in Intensive Care Units	Number of Deaths Attributable to COVID-19
Public health expenditures ^§^	0.54 *	−0.72 ^#^	−0.71 ^#^	−0.60 **	−0.72 ^#^
Mean seasonal temperature	0.28	−0.64 **	−0.64 **	−0.64 **	−0.55 **
Delay in applying the lockdown	−0.51 *	0.45 *	0.46 *	0.16	0.50 *
Import–export with China	−0.21	0.46 *	0.46 *	0.11	0.63 **
International air traffic	−0.18	0.17	0.17	−0.07	0.29
Mortality from CVD	0.61 **	−0.53 *	−0.26	−0.32	−0.28
Mortality from respiratory diseases	0.16	0.26	0.26	0.21	0.22

Influenza vaccination coverage rate: vaccination coverage rate of subjects aged 65 and over; CVD: cardiovascular diseases; ^§^ percentage of health expenditures with respect to the regional Gross Domestic Product. * *p* < 0.05; ** *p* < 0.01; ^#^ *p* < 0.0001.

**Table 2:** Multivariable relationships between regional influenza vaccination coverage rate in the Italian population aged 65 and over and COVID-19 outcomes.

Regional Features/Variables	SARS-CoV-2 Seroprevalence		Patients Hospitalized with Symptoms		Patients Hospitalized in Intensive Care Units		Number of Deaths Attributable to COVID-19	
	Beta [95% C. I.]	*p*	Beta [95% C. I.]	*p*	Beta [95% C. I.]	*p*	Beta [95% C. I.]	*p*
Influenza vaccination coverage rate	−130 (−198; −62)	0.001	−4.16 (−6.27; −2.05)	0.001	−0.58 (−1.05; −0.12)	0.017	−3.29 (−5.66; −0.93)	0.01
Public health expenditures *	−224 (−616; 168)	0.24	−3.56 (−15.73; 8.6)	0.54	−1.94 (−4.59; 0.71)	0.14	−7.90 (−21.53; 5.72)	0.23
Mean seasonal temperature	−123 (−359; 113)	0.28	−6.32 (−13.64; 1.01)	0.09	−0.73 (−2.33; 0.87)	0.34	−2.40 (−10.60; 5.80)	0.54
Delay in applying the lockdown	−70.8 (−145.1; 3.6)	0.06	−0.77 (−3.08; 1.54)	0.49	−0.45 (−0.96; 0.05)	0.07	−1.28 (−3.86; 1.30)	0.31
Import–export with China	0.66 (0.42; 0.90)	<0.0001	0.010 (0.001; 0.016)	0.032	0.000 (−0.002; 0.001)	0.87	0.01 (0.01; 0.02)	0.004
Mortality from CVD	652 (−88; 1392)	0.08	26.1 (3.1; 49.1)	0.029	1.35 (−3.66; 6.36)	0.57	22.7 (−3.0; 48.5)	0.08

* Percentage of health expenditures with respect to the regional Gross Domestic Product; CVD: cardiovascular diseases.

## Supplementary Materials

The following are available online at https://www.mdpi.com/2076-393X/8/3/535/s1, Table S1: Value of influenza vaccination coverage rate, of the four COVID-19 outcomes and of seven possible confounders used in the study (regional aggregate data), Table S2: R^2^ value before and after adjustment for confounders. Figure S1: Extrapolated values of influenza vaccine coverage rate used in this analysis.
