# Supplementary material for: Relationship between Influenza Vaccination Coverage Rate and COVID-19 Outbreak: An Italian Ecological Study
Source: Vaccines (Basel). 2020 Sep 16;8(3):535. doi: 10.3390/vaccines8030535 (PMC7563271; doi:10.3390/vaccines8030535)
Supplement: Supplementary file 1 [file vaccines-08-00535-s001.pdf]

Article

Relationship between Influenza Vaccination Coverage Rate and COVID-19 Outbreak: An Italian Ecological Study

Mauro Amato [1], José Pablo Werba [1], Beatrice Frigerio [1], Daniela Coggi [1,2], Daniela Sansaro [1], Alessio Ravani [1], Palma Ferrante [1], Fabrizio Veglia [1], Elena Tremoli [1] and Damiano Baldassarre [1,3,*]

1 Centro Cardiologico Monzino, IRCCS, 20138 Milan, Italy; mauro.amato@ccfm.it (M.A.), pablo.werba@ccfm.it (J.P.W.); beatrice.frigerio@ccfm.it (B.F.); daniela.coggi@ccfm.it (D.C.); daniela.sansaro@ccfm.it (D.S.); alessio.ravani@ccfm.it (A.R.); ferrante.palma@cardiologicomonzino.it (P.F.); fabrizio.veglia@ccfm.it (F.V.); elena.tremoli@ccfm.it (E.T.)

2 Dipartimento di Scienze Farmacologiche e Biomolecolari, Università degli Studi di Milano, 20133 Milan, Italy

3 Department of Medical Biotechnology and Translational Medicine, Università degli Studi di Milano, 20129 Milan, Italy

* Correspondence: damiano.baldassarre@unimi.it; Tel.: +39-02-580-02253

Received: 19 August 2020; Accepted: 12 September 2020; Published: 16 September 2020

Table S1. Value of influenza vaccination coverage rate, of the four COVID-19 outcomes and of seven possible confounders used in the study (regional aggregate data)

Regions/AP	Influenza vaccination coverage rate (%)	SARS-CoV-2 seroprevalence *	Patients hospitalized with symptoms*	Patients hospitalized in Intensive Care Units*	Number of deaths attributable to COVID-19*	Public Health Expenditure § (%)	Mean seasonal temperature (°C)	Delay in applying the lockdown (days)	Regional import-export with China (millions of Euros)	International air traffic (number of subjects)	Mortality from cardiovascular diseases*	Mortality from respiratory diseases*
Bozen (A.P.)	37	3300	91	17	55	5.8	5.7	28	94	0	3.02	0.62
Aosta Valley	45	4000	150	35	114	5.95	5.9	19	40	0	3.63	1.11
Sardinia	47	300	10	3	8	10.1	14.1	19	17	500	3.10	0.74
Lombardy	49	7500	132	14	161	4.95	7.7	32	7953	11433	3.24	0.82
Piedmont	50	3000	115	11	89	6.43	6.5	38	1956	644	4.24	1.06
Liguria	51	3100	89	12	95	6.65	11.7	33	324	168	4.96	1.21
Marche	53	2700	76	12	65	7.32	9.8	27	573	102	4.32	0.99
Lazio	53	1000	27	4	13	5.67	10.1	34	767	11109	3.65	0.88
Trento (A.P.)	55	3100	74	16	86	6.18	5.7	21	99	0	3.18	0.63
Emilia Romagna	56	2800	89	9	93	5.65	8.1	32	2653	2402	3.86	1.01
Veneto	56	1900	37	7	39	5.65	7.2	38	2677	3775	3.47	0.80
Abruzzo	56	1500	37	7	32	7.9	9.4	19	170	97	4.62	0.91

Sicily	56	300	12	2	6	10.9	12.4	21	198	1344	4.24	0.82
Tuscany	58	1000	32	9	28	6.49	10.3	30	1263	1706	4.09	1.02
Puglia	58	900	19	5	13	10.4	12.2	22	411	806	3.62	0.83
Friuli Venezia Giulia	60	1000	31	7	28	6.71	8.4	23	507	99	4.22	1.11
Campania	62	700	17	4	7	9.93	11.2	21	935	1897	3.83	0.71
Calabria	64	600	10	2	5	11.3	12.2	17	31	121	4.42	0.78
Umbria	65	900	22	7	9	8.14	9.2	18	141	44	4.39	1.05
Molise	66	700	21	4	7	10.4	8.3	8	44	0	5.24	0.90
Basilicata	67	800	14	4	5	9.37	8.8	10	27	0	4.48	1.00
**Average**	55.4	1957	52.6	9.10	45.6	7.71	9.33	24.3	994	1726	3.99	0.90
**Range (Min; Max)**	(37; 67)	(300; 7500)	(10; 150)	(2; 35)	(5; 161)	(4.95; 11.3)	(5.7; 14.1)	(8; 38)	(17; 7953)	(0; 11433)	(3.02; 5.24)	(0.62; 1.21)

The last two rows show the average and the range values of the variables considered; * cases/100,000 inhabitants; § Public health expenditure as % of regional Gross Domestic Product (GDP).

Table S2. R^2 value before and after adjustment for confounders*.

COVID-19 Outcome	Unadjusted R^2	R^2 after adjustment for confounders	R^2 change (%)
SARS-CoV-2 seroprevalence	0.34	0.88	159
Patients hospitalized with symptoms	0.45	0.82	82
Patients hospitalized in Intensive Care Units	0.36	0.70	94
Number of deaths attributable to COVID-19	0.33	0.78	136

*percentage of health expenditures with respect to the regional Gross Domestic Product, mean seasonal temperature delay in applying the lockdown, import-export with China and mortality from cardiovascular disease.

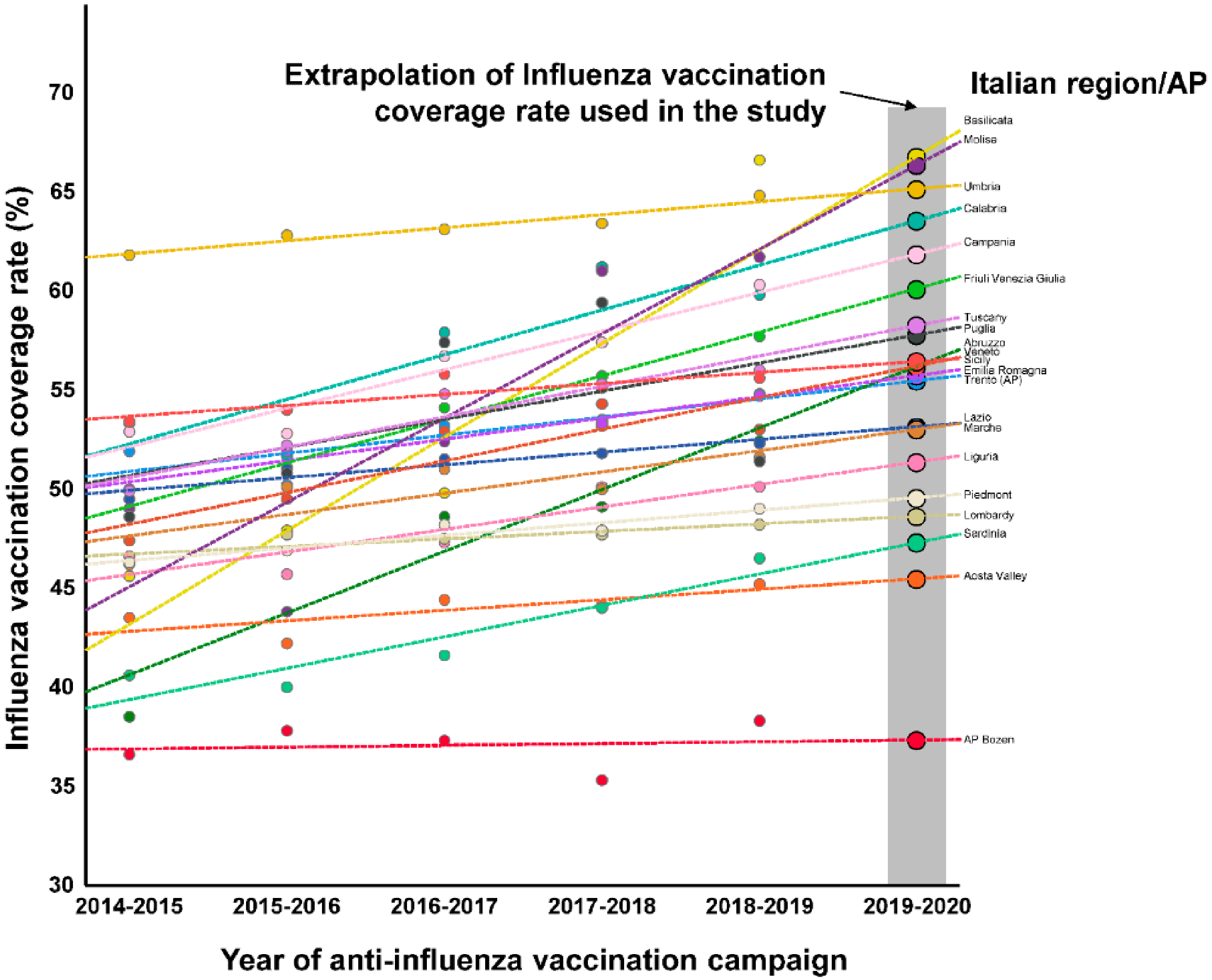

Figure S1. Extrapolated values of influenza vaccine coverage rate used in this analysis.
